# Les fractures luxations du cotyle: prise en charge et pronostic à long terme; étude rétrospective portant sur 40 cas

**DOI:** 10.11604/pamj.2014.19.90.5101

**Published:** 2014-09-26

**Authors:** Aniss Chagou, Ismail Hmouri, Abdelkarim Rhanim, Abdou Lahlou, Mohammed Saleh Berrada, Moradh Yaacoubi

**Affiliations:** 1 Service de Traumatologie-Orthopédie, Centre Hospitalier Universitaire de Rabat, Université Mohammed V, Rabat, Maroc

**Keywords:** Fracture-luxation, cotyle, traitement orthopédique, traitement chirurgical, fracture-dislocations, acetabulum, orthopedic treatment, surgical treatment

## Abstract

Les fractures luxations du cotyle sont le plus souvent dues à des traumatismes de haute énergie. Elles constituent une urgence thérapeutique, l'association de la luxation à une fracture du cotyle fait apparaître la question du choix thérapeutique entre traitement orthopédique et traitement médical. L'objectif de l’étude est de mettre le point sur l'aspect thérapeutique dans ces lésions mais aussi leurs pronostics à long terme. Nous rapportons une étude rétrospective portant sur 40 cas colligés au service d'orthopédie du centre hospitalier universitaire de Rabat. Nous avons évalué les résultats de notre prise en charge mais aussi le pronostic à court et à long terme. Dans notre série, vingt cinq patients ont bénéficié d'un traitement orthopédique alors que les quinze restants ont été opérés, la voie d'abord la plus utilisée est la voie postérieure. Les résultats fonctionnels ont été évalués, après un recul de 3 à 8 ans, selon la cotation de Merle d'Aubigné. Nous avons obtenu 90% de résultats satisfaisants. La comparaison de nos résultats à ceux de la littérature montre que le résultat des traitements orthopédiques et chirurgicaux dépend essentiellement du type de fracture. Le pronostic à long terme reste imprévisible. La survenue des complications tardives telle que la nécrose céphalique et de l'arthrose reste toujours imprévisible, ce qui impose un suivi régulier et prolongé des patients.

## Introduction

Les fractures luxations du cotyle sont la résultante de traumatisme à haute énergie. Elles sont souvent associées à d'autres lésions générales ou régionales et doivent donc être prises en charge par une équipe pluridisciplinaire. Il s'agit d'une urgence thérapeutique qui impose la réduction de la luxation dans les plus brefs délais pour éviter la nécrose de la tête fémorale. Par ailleurs la question du choix thérapeutique entre traitement orthopédique et chirurgical s'impose du fait de l'association de la luxation à une fracture du cotyle. Le pronostic de ces lésions est imprévisible, la survenue de nécrose de la tête fémorale et de coxarthrose même si le traitement est adéquat. L'objectif de l’étude que nous rapportons est de mettre le point sur la prise en charge de ces fractures ainsi que leurs pronostics à long terme.

## Méthodes

Il s'agit d'une étude rétrospective portant sur 40 cas colligés au centre hospitalier universitaire de Rabat entre 2005 et 2011. Nous évaluons le résultat de notre prise en charge mais aussi de leur pronostic à court et à long terme. L’évaluation est clinique, radiologique et fonctionnelle après un recul de 3 à 8 ans.

## Résultats

L’âge moyen de nos patients est de 35 ans, avec des extrêmes allant de 17 à 79 ans. La tranche d’âge la plus touchée est celle comprise entre 20 et 50 ans, soit 75% avec une nette prédominance masculine de 92,5% des cas: 37 hommes contre 3 femmes. Nous notons également une légère prédominance de l'atteinte de la hanche droite 55%. Dans tous les cas que nous avons colligé, il s'agissait d'un traumatisme violent, le plus souvent un accident de la voie publique 34 cas (85%), réalisant le classique choc du « tableau de bord », suivi par les chutes 3 cas (7,5%) et les accidents du travail 3 cas (7,5%). Tous les patients ont bénéficié d'un bilan radiologique comportant un cliché de bassin face, un cliché ¾ alaire, et un cliché ¾ obturateur. Ce qui nous a permis de faire le diagnostic des lésions synthétisées dans le Tableau 1.

**Tableau 1:** Classification selon la lésion anatomopathologique et fréquence de ces lésions

Type anatomopathologique	Nombre de cas	Fréquence
Lux post + Fr. paroi post	25	62,5%
Lux post + Fr. colonne post	6	15%
Lux post + Fr. rebord cotyloïdien	2	5%
Lux post + Fr. transversale	3	7,5%
Lux post + Fr. complexe	3	7,5%
Lux centrale + Fr. transversale	1	2,5%

Nous avons souvent eu affaire à des patients polytraumatisés puisque 18 de ces patients présentaient d'autres lésions associées. Nous avons classé ces lésions en celles compromettant le pronostic vital où nous avons 4 cas de traumatisme crânien soit 10% des cas et 4 cas de traumatismes thoraciques soit 10%des cas. Les autres lésions ne compromettaient pas le pronostic vital, nous avions 3 cas de paralysie du sciatique poplité externe, 3 cas de fracture de fémur, 3 cas de fracture du tibia, 2 cas de fracture de métacarpiens, 1 cas de fracture du cadre obturateur et aileron sacré droit et 1 cas de fracture de la tête fémorale.

**Figure 1:**
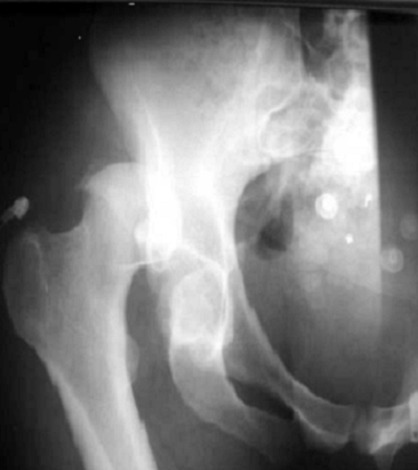
Radiographie de la hanche de face montrant une fracture de la paroi postérieure associée à une luxation de la tête fémorale

**Figure 2:**
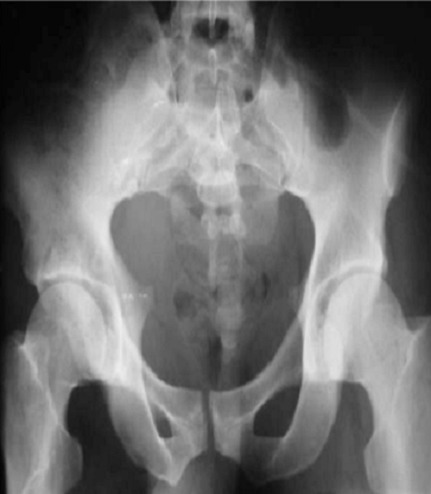
Radiographie du bassin de face après réduction de la luxation, le patient a été traité orthopédiquement

**Figure 3:**
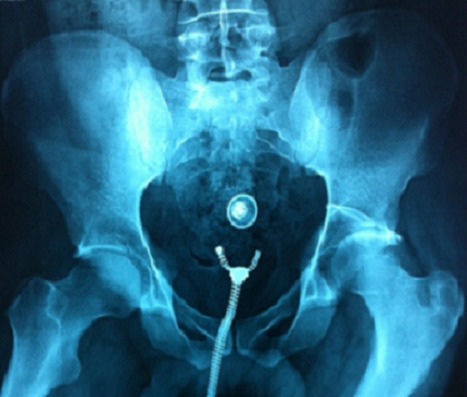
Radiographie bassin de face montrant une fracture luxation de la paroi postérieure

**Figure 4:**
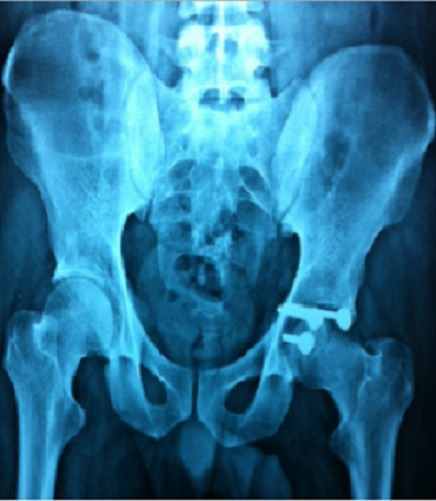
Contrôle postopératoire, traitement par vissage

Concernant le traitement, sur 40 cas, nous avons noté que 23 cas de fractures luxations ont été réduites dans les 24 heures, dix-sept entre 0 et 12 heures, six ente 12 et 24 heures, 2 après 24 heures et deux dans un délai non précisé. 7 cas de étaient irréductibles: l'incarcération osseuse empêchait la réintégration céphalique; 6 cas de réduction incoercible: la réduction était instable, elle se reproduisait immédiatement par manque de maintien postérieur ce qui a justifié la mise en place d'une traction provisoire en attendant le traitement définitif. Le traitement de la fracture articulaire a été orthopédique (Figure 1, Figure 2) dans 25 cas consistant en une traction trans-tibiale ou sus condylienne pour des durées allant de 15 à 45 jours. Il a été chirurgical (Figure 3, Figure 4) dans 15 cas (Tableau 2).

**Tableau 2:** Répartition des patients selon le traitement reçu, traitement orthopédique ou chirurgical

Traitement orthopédique	Traitement chirurgical
9 cas de fracture de la paroi postérieure	4 cas de luxations postérieures avec fracture de la colonne postérieure
2 cas de fracture de la colonne postérieure	3 cas de luxation postérieure avec fracture complexe du cotyle
4 cas de fractures transversales	2 cas de fractures luxations de la hanche négligées (15 jours et 19 jours)
2 cas de fracture du rebord cotyloïdien	-

**Figure 5:**
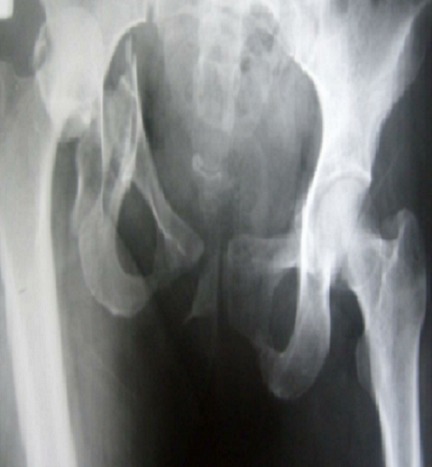
Radiographie du bassin de face montrant une fracture transversale du cotyle droit

**Figure 6:**
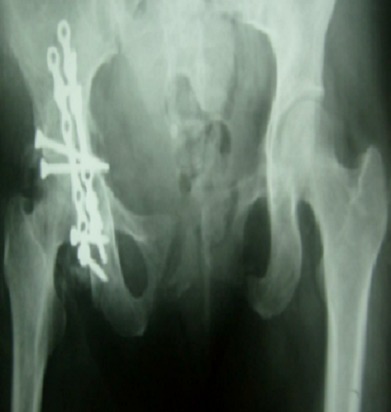
Radiographie de contrôle après traitement par double voie d'abord antérieure et postérieure

La voie d'abord utilisée est la voie postérieure de Kocher-Langenbeck sauf pour un patient où la voie postérieure de Kocher-Langenbeck a été combinée à une voie ilio-inguinale (Figure 5, Figure 6). L'ostéosynthèse a été réalisée soit par un vissage simple soit par des plaques vissées. Les résultats fonctionnels ont été évalués, après un recul de 3 à 8 ans, selon la cotation de Merle d'Aubigné [1]. Nous avons obtenu 90% de résultats satisfaisants: 14 cas de très bons résultats, 16 cas de bons résultats; 6 cas de moyens résultats, 4 cas de mauvais résultats avec une fréquence de 10%:1 cas de reluxation et 3 cas d'ossifications péri-articulaires. Nos résultats sont synthétisés dans le Tableau 3.

**Tableau 3:** Tableau synthétique rassemblant tous nos résultats en fonction du type de fracture, en fonction du traitement et comparaison des résultats selon le traitement

Résultats en fonction du type de la fracture luxation			
Type de fracture luxation	satisfaisants		Mauvais
Lux post + FR PP	23		2
Lux post + FR colonne post	6		-
Lux post + FR transversale	2		-
Lux post + FR du rebord cotyloïdien	2		1
Lux post + FR complexe	2		1
Lux centrale + FR transversale	1		-
**Résultats en fonction du traitement**			
Résultats	Nombre de cas	orthopédique	Chirurgical
Très bon	16	11	5
Bon	14	10	4
Moyen	6	2	4
Mauvais	4	2	2
**Comparaison des résultats selon le traitement**			
Traitement	Nombre de cas	Résultats satisfaisants	Mauvais résultats
Orthopédique	25	23 (92%)	2 (8%)
chirurgical	15	13 (86,66%)	2 (13,33%)

## Discussion

Les fracture-luxations de la hanche ont vu leurs fréquences augmenter avec la multiplication des accidents de la circulation, elle représente 36% de l'ensemble des fracture du cotyle selon Duquennoy [2], 38% selon Rafai [3] et 38% dans notre série. Cette lésion survient avec prédilection chez l'adulte jeune masculin [3, 4], les résultats de notre série vont dans le sens de cette constatation, ce qui peut être expliqué par la fréquence des accidents chez l'adulte jeune actif et surtout de sexe masculin. Cette fracture luxation est causée le plus souvent par des accidents de la voie publique [3, 4, 5], dans notre série nous avons 36 cas d'accidents de la voie publique ce qui représente 90%.

Le cliché de face et les deux clichés de ¾ permettent d'affirmer le diagnostic, de préciser le type de la luxation et de la fracture associée. Ils étaient systématiques chez tous nos patients. Le type lésionnel le plus fréquent est la luxation postérieure avec fracture de la paroi postérieure, avec une fréquence de 62,5%. La TDM affine l'analyse des dégâts articulaires et découvre de nombreuses lésions associées méconnues à la radiographie standard. Ainsi la TDM permet de mettre en évidence des corps étrangers intra-articulaires, de préciser les lésions osseuses et de découvrir d'autres lésions passées inaperçues en particulier sacro-iliaques, de même elle vérifie la congruence articulaire, et montre les impactions, les incarcérations et les encoches de la tête fémorale échappant à la radiographie standard [6]. Dans notre série, nous avons eu recours à la TDM dans 36 cas, et elle a objectivé en plus des lésions découvertes à la radiologie standard deux cas de fractures complexe de la paroi postérieure avec présence de multiples fragments osseux dans les parties molles adjacentes dont le nombre et le volume étaient invisibles sur les radiographies standards, une fracture de la paroi postérieure avec fracture du toit du cotyle, une fracture de la paroi postérieure avec un fragment osseux intra-articulaire et deux cas d’épanchement articulaire. Ces résultats confirment la nécessité de demander la TDM devant toute suspicion de fracture luxation de la hanche.

Les circonstances de survenue de la fracture luxation de la hanche lors d'un AVP expliquent la fréquente association à d'autres lésions locales et générales. Dans notre série, nous avons noté dans 45% des cas (18 cas) des lésions associées contre 73,6% des cas (38 cas) dans la série de Troncoso [7]. Les lésions graves crâniennes et thoraciques sont fréquentes dans les deux séries. Le traitement a pour but de récupérer la fonction de la hanche par la reconstruction anatomique des surfaces articulaires et leur contention stable et solide.

Dans notre série, le choix de ce traitement a été justifié dans certains cas du fait que la fracture était bénigne, et ne mettait pas en cause la stabilité de la réduction de la luxation, et dans d'autre cas du fait de la complexité des lésions cotyloïdiennes qui sont impossible à réduire et à contenir chirurgicalement. Pendant cette période, le patient commence la mobilisation passive de la hanche, il reprend la marche avec cannes-béquilles environs 3 mois après l'accident. Dans notre série, le traitement chirurgical a été utilisé dans 25 cas (62,5%), 13 fois d'emblée pour échec de la réduction orthopédique, et 12 fois pour persistance d'un déplacement fracturaire après réduction de la luxation. La voie d'abord postérieure de Kocher-Langenbeck a été presque la seule utilisée dans notre série par contre Kumar [8] sur 73 cas de fracture du cotyle, un abord simple a été utilisé dans 67 cas (92%), 41 cas ont été opéré par voie postérieure de Kocher-Langenbeck, 26 cas par voie ilio-inguinale, 5 cas ont nécessité la voie tri radiée, et un seul cas a été opéré par voie combinée antérieure et postérieure. L'ostéosynthèse a été soit un vissage simple, soit un vissage avec plaque vissée pour tous les auteurs.

Concernant les complications, nous avons relevé deux cas de surinfection de broches de traction. Troncoso [7], sur 38 cas, a relevé un cas d'ostéite par broche de traction, soit une fréquence de 2,6%. Par ailleurs, toutes les études [4–11] insistent sur le fait que la nécrose de la tête fémorale est favorisée par le retard de réduction de la luxation. Il semble pratiquement impossible de prévenir ou d’éviter la survenue de la nécrose fémorale car tout dépend du degré de l'atteinte vasculaire produite au moment du choc ou au moment de la réduction. Dans notre série, nous n'avons eu aucun cas de nécrose de la tête fémorale. Nous n'avons observé aucun cas d'arthrose dans notre série mais Il faut signaler que le recul dans nos observations est insuffisant pour prédire la fréquence réelle des complications tardives. Nous avons obtenus dans notre étude 90% de résultats satisfaisants ce qui concorde avec ceux de la littérature (Tableau 4).

**Tableau 4:** Comparaison du résultat global de notre série avec celui d'autres séries

	Très bon	bon	moyen	Mauvais
MERLE D'AUBIGNE [7]	11 (52%)	4 (20%	2 (10%)	3 (15%)
TRONCOSO [11]	10 (26%)	9 (24%)	8 (21%)	11 (29%)
JIRARI [2]	9 (20%)	16 (35%)	5 (11%)	15 (33%)
Notre série	14 (35%)	16 (40%)	6 (15%)	4 (10%)

## Conclusion

Les fractures luxations du cotyle sont la résultante de traumatisme à haute énergie, mettant en jeu le pronostic fonctionnel de la hanche et parfois le pronostic vital. Le scanner doit être demandé systématiquement car il précise bien les lésions locales et montre d'autres lésions associées. La survenue des complications tardives telle que la nécrose céphalique et de l'arthrose reste toujours imprévisible, ce qui impose un suivi régulier et prolongé des patients.
